# Risk factors for peripheral intravascular catheter-related phlebitis in critically ill patients: analysis of 3429 catheters from 23 Japanese intensive care units

**DOI:** 10.1186/s13613-022-01009-5

**Published:** 2022-04-08

**Authors:** Hideto Yasuda, Claire M. Rickard, Nicole Marsh, Ryohei Yamamoto, Yuki Kotani, Yuki Kishihara, Natsuki Kondo, Kosuke Sekine, Nobuaki Shime, Keita Morikane, Takayuki Abe, the AMOR-NUS study group

**Affiliations:** 1 Department of Emergency and Critical Care Medicine, Jichi Medical University Saimata Medical Center, 1-847, Amanuma-cho, Oomiya-ku, Saitama-shi, Saitama, 330-8503 Japan; 2 Department of Clinical Research Education and Training Unit, Keio University Hospital Clinical and Translational Research Center (CTR), Tokyo, Japan; 3 School of Nursing, Midwifery and Social Work, UQ Centre for Clinical Research, The University of Queensland, Brisbane, QLD Australia; 4 School of Nursing and Midwifery, and Alliance for Vascular Access Teaching and Research, Griffith University, Nathan, QLD Australia; 5 Herston Infectious Diseases Institute; Nursing and Midwifery Research Centre, Royal Brisbane and Women’s Hospital, Metro North Hospital and Health Service, Herston, QLD Australia; 6 Department of Healthcare Epidemiology, School of Public Health in the Graduate School of Medicine, Kyoto University, Kyoto, Japan; 7 Department of Intensive Care Medicine, Kameda Medical Center, Chiba, Japan; 8 Department of Intensive Care Medicine, Chiba Emergency Medical Center, Chiba-shi, Japan; 9 Department of Medical Engineer, Kameda Medical Center, Chiba, Japan; 10 Department of Emergency and Critical Care Medicine, Graduate School of Biomedical and Health Sciences, Hiroshima University, Hiroshima, Japan; 11 Division of Clinical Laboratory and Infection Control, Yamagata University Hospital, Yamagata, Japan; 12 Biostatistics, Clinical and Translational Research Center, Keio University School of Medicine, Tokyo, Japan; 13 School of Data Science, Yokohama City University, Kanagawa, Japan

**Keywords:** Catheter, Catheter-related infections, Critically ill patient, Risk factors, Intensive care unit, Catheterization, Peripheral, Phlebitis

## Abstract

**Background:**

Phlebitis is an important complication occurring in patients with peripheral intravascular catheters (PIVCs). The risk factors for phlebitis in the intensive care unit (ICU) was examined.

**Methods:**

A secondary analysis of a prospective multicenter cohort study was conducted, involving 23 ICUs in Japan—the AMOR–VENUS study. Consecutive patients aged ≥ 18 years admitted to the ICU with newly inserted PIVCs after ICU admission were enrolled. Characteristics of the ICU, patients, PIVCs, and the drugs administered via PIVCs were recorded. A marginal Cox regression model was used to identify the risk factors associated with phlebitis.

**Results:**

A total of 2741 consecutive patients from 23 ICUs were reviewed for eligibility, resulting in 1359 patients and 3429 PIVCs being included in the analysis population. The median dwell time was 46.2 h (95% confidence interval [CI], 21.3–82.9). Phlebitis occurred in 9.1% (95% CI, 8.2–10.1%) of catheters (3.5 cases/100 catheter days). The multivariate analysis revealed that the only factors that increased the risk of developing phlebitis were drugs administered intravenously. This study included 26 drugs, and 4 were associated with increased phlebitis: nicardipine (HR, 1.85; 95% CI, 1.29–2.66), noradrenaline (HR, 2.42; 95% CI, 1.40–4.20), amiodarone (HR, 3.67; 95% CI, 1.75–7.71) and levetiracetam (HR, 5.65; 95% CI, 2.80–11.4). Alternatively, factors significantly associated with a reduced risk of phlebitis were: standardized drug administration measures in the ICU (HR, 0.35; 95% CI, 0.17–0.76), 30≤ BMI (HR, 0.43; 95% CI, 0.20–0.95), catheter inserted by a doctor as nurse reference (HR, 0.55; 95% CI, 0.32–0.94), and upper arm insertion site as forearm reference (HR, 0.52; 95% CI, 0.32–0.85). The nitroglycerin was associated with a reduced phlebitis risk (HR, 0.22; 95% CI, 0.05–0.92).

**Conclusion:**

Various factors are involved in the development of phlebitis caused by PIVCs in critically ill patients, including institutional, patient, catheter, and drug-induced factors, indicating the need for appropriate device selection or models of care in the ICU.

*Trial registration:* UMIN-CTR, the Japanese clinical trial registry (registration number: UMIN000028019, July 1, 2017).

**Supplementary Information:**

The online version contains supplementary material available at 10.1186/s13613-022-01009-5.

## Background

Peripheral intravascular catheters (PIVCs) are essential invasive medical devices in the intensive care unit (ICU) [1]. Complications associated with PIVCs, especially phlebitis (irritation or inflammation of the vein wall), are common, occurring at a high rate and reaching one-quarter (23.8%) of catheterized patients [2]. Phlebitis not only causes pain, anxiety, and interruption of treatment, but can also result in serious complications such as skin necrosis and infective endocarditis [3–5].

Previous studies in general wards have identified risk factors for phlebitis, including insertion site, catheter design, material, size, dressing material used, type of medication administered, and the number of catheter days [6–15]. However, risk factors for phlebitis are yet to be explored in ICU [9, 16]. Although the PIVC insertion frequency in the ICU may be higher than in the general wards, the duration of ICU stay may be shorter than that in the general wards. Furthermore, types of intravenous drugs administered through PIVCs in critically ill patients may differ from those in general wards, and these drugs may be an important risk factor for catheter-related phlebitis in the ICU. The differences between general wards and intensive care units may influence phlebitis risk factors. However, few adequate preventive measures have been taken due to the lack of appropriate information on the epidemiology and risk factors of phlebitis in critically ill patients.

The AMOR–VENUS study demonstrated the epidemiology of the use of PIVCs and the incidence or occurrence of phlebitis and complications in critically ill patients [2]. In this study, the diagnostic criteria for phlebitis were in accordance with the criteria presented by the American Infusion Nurses Society (INS) [17] (Additional file 1: Table S1). A secondary analysis was conducted using the database in this study to identify the risk factors for phlebitis in critically ill patients.

## Methods

### Study design and setting

This study was conducted using the AMOR–VENUS database. This previous study was a prospective multicenter cohort study that involved 22 institutions and 23 ICUs in Japan between January 1, 2018, and March 31, 2018 [2]. The AMOR–VENUS study was pre-registered at UMIN-CTR under the Japanese clinical trial registry (registration number: UMIN000028019) and was approved by the Institutional Review Board or Medical Ethics Committee of each study institution. The protocol of the AMOR–VENUS study included the epidemiological study of PIVC-induced phlebitis in intensive care and a study of risk factors for phlebitis. However, the epidemiological information and the study of risk factors were presented as separate studies to clarify the discussion points due to the vast amount of epidemiological information. A new ethical review was waived for this study since the post hoc analysis using the AMOR–VENUS database had already been approved by the AMOR–VENUS ethical review. This study was reported in accordance with the Strengthening Reporting of Observational Studies in Epidemiology (STROBE) guidelines [18] (see Additional file 2).

### Study participants and included PIVCs

The AMOR–VENUS study database included all consecutive patients aged ≥ 18 years with PIVCs inserted during ICU admission. Only PIVCs that were newly inserted after ICU admission were included in this study to avoid immortal time bias, since detailed drug information administered through catheters is necessary for the analysis of this study. The inclusion and exclusion criteria details are described in the AMOR–VENUS study paper reported earlier [2].

### Data collection

The following information was collected in this study from the AMOR–VENUS study database: ICU characteristics (provision of standardized drug administration measures in the ICU, and provision of education on venous catheter management for nurses), patient characteristics (age, gender, body height, body weight, APACHE II, SAPS II, SOFA, Charlson comorbidity index, ICU admission routes, type of admission to ICU, ICU admission category, presence of sepsis at ICU admission, and presence of mechanical ventilation), PIVC characteristics (medical staff inserting the catheter, inserted site, catheter materials, catheter gauges, skin antiseptics, use of ultrasonography, number of trials for insertion, difficulties with the insertions, types of gloves, dressing methods, infection during catheter dwell, and duration of catheter dwell), information on the drugs administered via PIVCs during ICU stay, and the outcome of phlebitis. Details of the collected data in the original study are in Additional file 1.

### Study outcomes

The primary outcome was phlebitis defined using the Phlebitis Scale developed by the INS [17] (see Additional file 1: Tables S1 and S2). The primary objective was to identify the risk factors for phlebitis using explanatory analyses. Detailed information on the definition of phlebitis and evaluation methods was reported in the previously published AMOR–VENUS study and described in Additional file 1 of this paper. Assessors were blinded to all clinical characteristics with the exception of the six criteria necessary to diagnose phlebitis.

### Statistical methods

Patient and catheter characteristics were presented as means with standard deviations (SDs) or medians with interquartile ranges (IQRs) for the continuous variables and as percentages for the categorical variables. The association between the time to occurrence of phlebitis and risk factors using multivariable marginal Cox regression analyses was assessed to take into account the within-patient and within-institution correlations between the catheters. The time zero of the marginal COX regression model was set to the time of PIVC insertion in the ICU. The censoring was defined as removal of PIVC or ICU discharge if the patient left the ICU with the PIVC inserted. A Schoenfeld’s residual test was used to test the proportional hazards assumption in the Cox proportional hazards model. In this primary multiple regression model, 40 variables were included from four variable levels selected based on a priori knowledge and clinical perspectives: ICU characteristics (presence of education on venous catheter management for nurses, and standardized drug administration measures in the ICU), patient-level variables (age, gender, body mass index, and APACHE II), catheter-level variables (medical staff inserting the catheter, number of trials for insertion, use of ultrasonography, catheter insertion site, catheter gauge, type of dressing, catheter material, and presence of infections during catheter dwell), and drug-level variables (fentanyl, heparin, fat, nicardipine, dexmedetomidine, ampicillin/sulbactam, albumin, paracetamol, potassium, meropenem, steroid, ceftriaxone, vancomycin, magnesium, peripheral parenteral nutrition, phosphorus, noradrenaline, carperitide, midazolam, nitroglycerin, dobutamine, cefmetazole, amiodarone, cefepime, levetiracetam, and landiolol). The standardized drug administration measures in the ICU in this study were defined according to documented standard operating procedures for drug administration supervised by a pharmacist at the relevant institution, which included the drug’s composition, choice of administration route, administration rate, and contraindications to compounding. Spline curves were drawn to evaluate whether the continuous variables (age, body mass index, and APACHE II) had a linear effect on phlebitis, and whether they were judged not to be linear, cutoff values were set with reference to the spline curves and treated as categorical variables. The drugs were included in the multivariable model as binary data. The types of drugs included in the multivariable model were limited to those administered at a percentage more frequent than 5% of all PIVCs with a phlebitis incidence of at least 1%. Multivariate analysis was performed only for the complete cases without multiple imputations when missing values were found in the factors included in the multivariate analysis.

The multicollinearity between each factor was evaluated using a variance inflation factor, with a value of 10 or more being considered to be associated with multicollinearity. When multicollinearity was suggested, one of the variables was excluded from the model. A multivariate analysis was also performed using backward selection methods as a sensitivity analysis. The analyses were performed using JMP V.10.0 and SAS version 9.4 (SAS Inc., Cary, NC).

## Results

### Participating patients and PIVCs

**Fig. 1:**
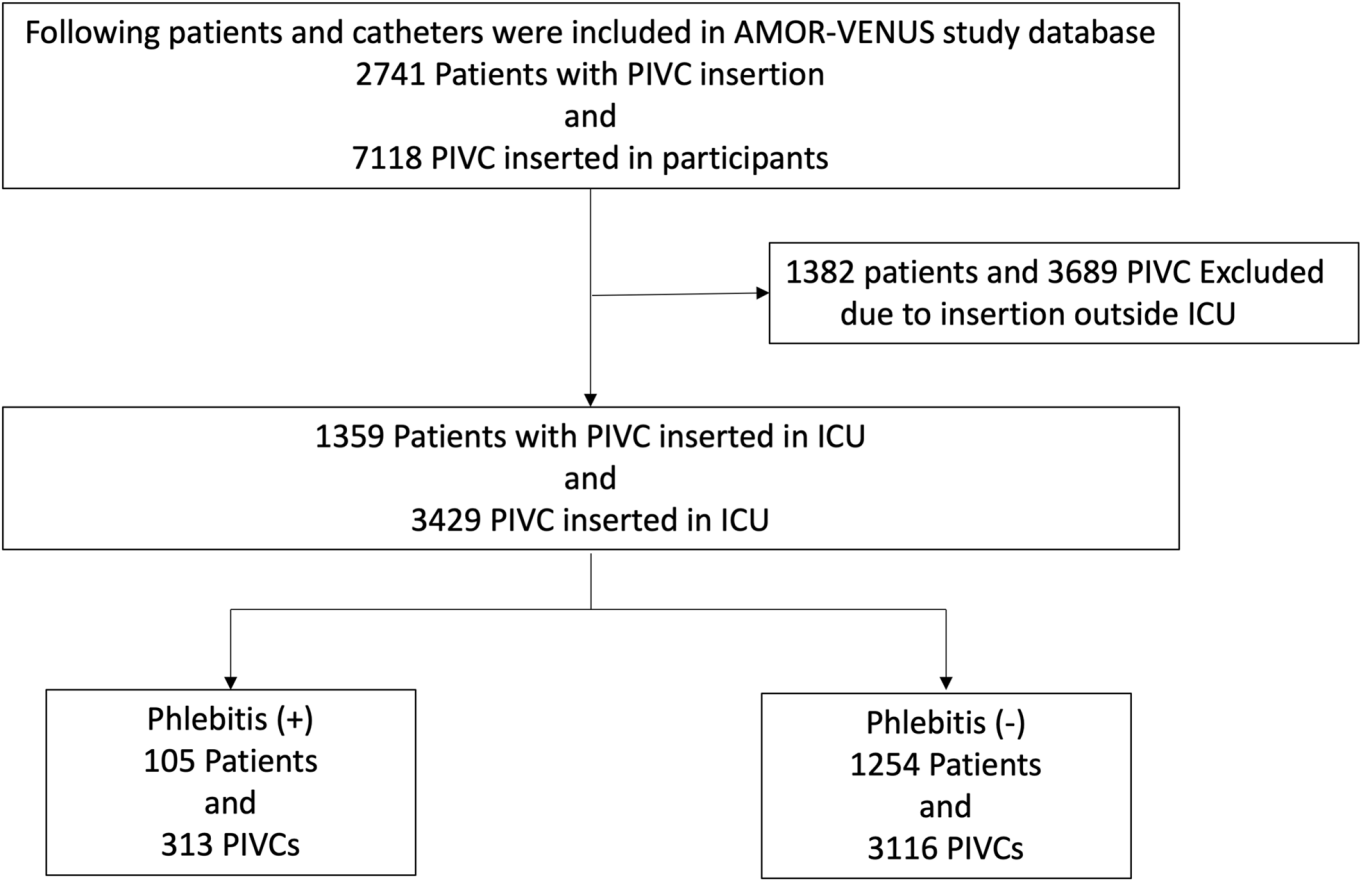
Patient flowchart

A total of 2741 patients and 7118 PIVCs from 23 ICUs were included in the AMOR–VENUS study database (Fig. 1). Of the 7118 PIVCs, 1382 patients and 3689 PIVCs were excluded due to insertion outside ICU. Finally, 1359 patients and 3429 PIVCs were analyzed (Fig. 1).

### Incidence of phlebitis

Phlebitis occurred in 313 of 3429 PIVCs (9.1%; 95% CI 8.2–10.1%) (see Additional file 1: Table S3). The incidence rate of phlebitis per 100 catheter insertion days was 3.5 (95% CI 3.1–3.9) of PIVCs. Details of the characteristics of phlebitis are shown in Additional file 1: Table S4.

### Patients’ characteristics and PIVCs with phlebitis

Table 1 shows the characteristics of patients with and without PIVC phlebitis at ICU admission. The mean ± SD age of the study population was 66.4 ± 15.8 years. Most patients (72.0%) were admitted to the ICU from the operating room (59.0% for elective operation and 13.0% for emergency operation), followed by the emergency room ([ER] 18.8%). Sepsis or septic shock accounted for 8.4% of the cases. The patients who developed phlebitis had a higher disease severity than those who did not (APACHE II, 16.9 ± 6.3 vs. 14.7 ± 7.2). They were more frequently admitted to the ICU via the general ward (12.4% vs. 6.7%) and were more frequently diagnosed with sepsis and septic shock at ICU admission (14.3% vs. 8.0%). On the other hand, the rate of elective postoperative status was lower in patients with phlebitis (35.2% vs. 61.0%).

**Table 1:** Patient characteristics at ICU admission with or without phlebitis

Variables	All patients *N* = 1359	Phlebitis ( +) *N* = 105 (7.7%)	Phlebitis (–) *N* = 1254 (92.3%)	*p* value
Age, mean (SD), years	66.4 (15.8)	70.9 (13.2)	66.1 (16.0)	0.003
Gender, male (*n*, %)	816 (60.0%)	63 (60.0%)	753 (60.1%)	0.99
Body height, mean (SD), cm^a^	161 (9.9)	161 (9.8)	161 (9.9)	0.99
Body weight, mean (SD), kg^b^	59.1 (14.2)	57.9 (14.0)	59.2 (14.2)	0.37
BMI, mean (SD)^a^	22.8 (4.3)	22.3 (4.3)	22.8 (4.3)	0.24
APACHE II, mean (SD)	14.8 (7.2)	16.9 (6.3)	14.7 (7.2)	0.003
SAPS II, mean (SD)	32.2 (18.5)	37.3 (14.6)	31.7 (18.7)	0.003
SOFA, mean (SD)	4.8 (3.4)	5.6 (3.3)	4.7 (3.4)	0.007
Charlson comorbidity index, mean (SD)	4.3 (2.6)	4.5 (2.5)	4.3 (2.6)	0.38
ICU admission from (*n*, %)				
Operation room	979 (72.0%)	58 (55.2%)	921 (73.5%)	< 0.0001
Emergency room	256 (18.8%)	29 (12.4%)	227 (18.1%)	0.02
General ward	97 (7.1%)	13 (12.4%)	84 (6.7%)	0.03
Outpatients	11 (0.8%)	2 (1.9%)	9 (0.7%)	0.19
Transfer from other hospital	16 (1.2%)	3 (2.9%)	13 (1.0%)	0.10
Type of admission to ICU (*n*, %)				
Elective surgical	802 (59.0%)	37 (35.2%)	765 (61.0%)	< 0.0001
Emergency surgical	177 (13.0%)	21 (20.0%)	156 (12.4%)	0.03
Medical	380 (28.0%)	47 (44.8%)	333 (26.6%)	< 0.0001
ICU admission category (*n*, %)				
Cardiology	438 (32.2%)	35 (33.3%)	403 (32.1%)	0.80
Pulmonary	171 (12.6%)	15 (14.3%)	156 (12.4%)	0.58
Gastrointestinal	295 (21.7%)	13 (12.4%)	282 (22.5%)	0.02
Neurology	127 (9.3%)	17 (16.2%)	110 (8.8%)	0.01
Trauma	47 (3.5%)	4 (3.8%)	43 (3.4%)	0.84
Urology	44 (3.2%)	3 (2.9%)	41 (3.3%)	0.82
Gynecology	37 (2.7%)	1 (1.0%)	36 (2.9%)	0.25
Skin/tissue	31 (2.3%)	1 (1.0%)	30 (2.4%)	0.34
Others	133 (9.8%)	12 (11.4%)	121 (9.7%)	0.56
Sepsis at ICU admission (*n*, %)				
Sepsis	48 (3.5%)	7 (6.7%)	41 (3.3%)	0.07
Septic shock	67 (4.9%)	8 (7.6%)	59 (4.8%)	0.19
Mechanical ventilation within 24 h after admission to ICU (n, %) ^c)^				
Non-invasive ventilation	55 (4.1%)	6 (5.8%)	49 (4.0%)	0.37
Invasive ventilation	343 (25.4%)	43 (41.8%)	300 (24.4%)	0.0001

*APACHE* Acute Physiology AND Chronic Health Evaluation, *BMI* body mass index, *ICU* intensive care unit, *SAPS* simplified acute physiology score, *PIVC* peripheral intravenous catheter, *SD* standard deviation, *SOFA* sequential organ failure assessment

Missing data: a) *n* = 2, b) missing data: n = 1, c) missing data: n = 27

Table 2 presents the PIVC characteristics with or without phlebitis. The most frequently used insertion site was the forearm (53.9%), followed by the back of the hand (14.8%). Of the 3429 PIVCs, 2102 (61.4%) were removed before ICU discharge, with a median PIVC insertion duration of 46.2 h and an interquartile range (IQR) of 21.3–82.9 h. In terms of the insertion site, the incidence of phlebitis was high with the catheters inserted in the forearm (60.7% with phlebitis vs. 53.3% without phlebitis) and low in the catheters inserted in the hand (10.5% with phlebitis vs. 15.2% without phlebitis). In addition, the number of phlebitis cases differed depending on the catheter material: PEU-Vialon material was less common in catheters with phlebitis (26.5% with phlebitis vs. 32.2% without phlebitis), but polyurethane material was more common (34.8% with phlebitis vs. 27.9% without phlebitis).

**Table 2:** All PIVC characteristics during insertion with or without phlebitis

Variables	All catheter *N* = 3429	Phlebitis ( +) *N* = 313	Phlebitis (−) *N* = 3116	*P* value
Catheter inserted by (*n*, %)^a^				
Doctor	287/2663 (10.8%)	21/246 (8.0%)	266/2417 (11.0%)	0.13
Nurse	2393/2663 (89.9%)	243/246 (92.0%)	2150/2417 (89.0%)	0.12
Medical technologist	1/2663 (0.04%)	0/246 (0%)	1/2417 (0.04%)	0.74
Inserted site (*n*, %)				
Upper arm	356/3429 (10.4%)	22/313 (7.0%)	334/3116 (10.7%)	0.04
Forearm	1849/3429 (53.9%)	190/313 (60.7%)	1659/3116 (53.2%)	0.01
Elbow	163/3429 (4.8%)	15/313 (4.8%)	148/3116 (4.8%)	0.97
Wrist	162/3429 (4.7%)	11/313 (3.5%)	151/3116 (4.9%)	0.29
Hand	507/3429 (14.8%)	33/313 (10.5%)	474/3116 (15.2%)	0.03
Lower leg	225/3429 (6.6%)	23/313 (7.4%)	202/3116 (6.5%)	0.55
Dorsal foot	137/3429 (4.0%)	17/313 (5.4%)	120/3116 (3.9%)	0.17
Others	30/3429 (0.9%)	2/313 (0.6%)	28/3116 (0.9%)	0.46
Catheter material				
PEU-Vialon*	1087/3116 (31.7%)	83/313 (26.5%)	1004/3116 (32.2%)	0.04
Polyurethane	978/3116 (28.5%)	109/313 (34.8%)	869/3116 (27.9%)	0.01
Polyethylene	0/3116 (0%)	0/313 (0%)	0/3116 (0%)	–
Tetrafluoroethylene	1292/3116 (37.7%)	111/313 (35.5%)	1181/3116 (37.9%)	0.40
Others	72/3116 (2.1%)	10/313 (3.2%)	62/3116 (2.0%)	0.16
Catheter gauge (n, %)^b^				
14G	1/3368 (0.03%)	0/308 (0%)	1/3060 (0.03%)	0.75
16G	74/3368 (2.2%)	9/308 (2.9%)	65/3060 (2.1%)	0.36
18G	89/3368 (2.6%)	8/308 (2.6%)	81/3060 (2.7%)	0.96
20G	888/3368 (26.4%)	56/308 (18.2%)	832/3060 (27.2%)	< 0.01
22G	2254/3368 (66.9%)	226/308 (73.4%)	2028/3060 (66.3%)	0.01
24G	62/3368 (1.8%)	9/308 (2.9%)	53/3060 (1.7%)	0.14
Antiseptic solution before catheterization (*n*, %)^c^				
None	8/2665 (0.3%)	1/260 (0.3%)	7/2405 (0.3%)	0.79
Alcohol	2599/2665 (97.5%)	254/260 (97.7%)	2345/240 (97.5%)	0.85
0.2% chlorhexidine alcohol	21/2665 (0.8%)	1/260 (0.3%)	20/2405 (0.8%)	0.44
0.5% chlorhexidine alcohol	15/2665 (0.6%)	3/260 (1.2%)	12/2405 (0.5%)	0.18
1.0% chlorhexidine alcohol	17/2665 (0.6%)	0/260 (0%)	17/2405 (0.7%)	0.17
10% povidone iodine	2/2665 (0.08%)	0/260 (0%)	2/2405 (0.08%)	0.64
Other	3/2665 (0.1%)	1/260 (0.3%)	2/2405 (0.08%)	0.17
Use of ultrasonography (*n*, %)^d^	58/2636 (2.2%)	4/260 (1.6%)	54/2376 (2.3%)	0.44
Number of trials for insertion (*n*, %)^e^				
1	2119/2619 (80.9%)	207/257 (80.6%)	1912/2362 (81.0%)	0.88
2	313/2619 (12.0%)	27/257 (10.5%)	286/2362 (12.1%)	0.45
3	130/2619 (5.0%)	18/257 (7.0%)	112/2362 (4.7%)	0.11
4	26/2619 (1.0%)	1/257 (0.4%)	25/2362 (1.1%)	0.30
5	15/2619 (0.6%)	1/257 (0.4%)	14/2362 (0.6%)	0.68
≥6	16/2619 (0.6%)	3/257 (1.2%)	13/2362 (0.6%)	0.23
Difficulties with the insertions (*n*, %)^f^				
Easy	1232/2594 (47.5%)	113/253 (44.7%)	1119/2341 (47.8%)	0.34
Slightly easy	772/2594 (29.8%)	76/253 (30.0%)	696/2341 (29.7%)	0.92
Slightly difficult	456/2594 (17.6%)	49/253 (19.4%)	407/2341 (17.4%)	0.43
Difficult	134/2594 (5.2%)	15/253 (5.9%)	119/2341 (5.1%)	0.56
Glove (*n*, %)^g^				
Sterile	19/2630 (0.7%)	0/259 (0%)	19/2371 (0.8%)	0.15
Non-sterile	2496/2630 (94.9%)	244/259 (94.2%)	2252/2371 (95.0%)	0.59
Nothing	115/2630 (4.4%)	15/259 (5.8%)	100/2371 (4.2%)	0.24
Dressing (*n*, %)^h^				
Chlorhexidine-impregnated dressing chrolehexidne	0/3396 (0%)	0/307 (0%)	0/3089 (0%)	-
Sterile polyurethane dressing	3327/3396 (98.0%)	298/307 (97.1%)	3029/3089 (98.1%)	0.24
Non-sterile polyurethane dressing polyuretane	60/3396 (1.8%)	8/307 (2.6%)	52/3089 (1.7%)	0.24
Gauze dressing	1/3396 (0.03%)	0/307 (0%)	1/3089 (0.03%)	0.75
Tape dressing	8/3396 (0.2%)	1/307 (0.3%)	7/3089 (0.2%)	0.73
Any infection during catheter dwell (*n*, %)	803/3429 (23.4%)	90/313 (28.8%)	713/3116 (22.9%)	0.02
Duration of catheter dwell, median (IQR), hour	46.2 (21.3–82.9)	37.0 (19.2–57.6)	44.8 (21.0–81.5)	< 0.01

*ICU* intensive care unit, *IQR* interquartile range, *PIVC* peripheral intravenous catheter

*ER* emergency room, *OR* operation room

*PEU-Vialon: polyetherurethane without leachable additives missing data: a) n = 699, b) missing data: n = 56, c) missing data: n = 711, d) missing data: n = 740, e) missing data: n = 754, f) missing data: n = 775, g) missing data: n = 745, h) missing data: *n* = 30

### Comparisons of characteristics of PIVC and Drugs between with and without phlebitis

Table 3 shows the drug characteristics included in the multivariable model. More than 300 drugs were administered to the included patients, and 26 were administered in at least 5% of patients, with a phlebitis frequency of ≥ 1%. As outlined in Table 3, fentanyl was the most commonly administered drug (13.5%), followed by heparin (9.7%) and nicardipine (9.0%). As for the incidence of phlebitis, levetiracetam had the highest (26.8%), followed by amiodarone (22.0%), noradrenaline (21.6%), and midazolam (20.0%). Statistical analyses show that nicardipine, noradrenaline, and potassium were associated with an increased incidence of phlebitis. Levetiracetam was associated with an increased incidence of phlebitis, although it was administered less frequently than the others.

**Table 3:** Comparison of the drugs administrated via PIVC with or without phlebitis

	No. of catheters with drug administration *N* = 3429	Proportion of phlebitis within drug (%)	Phlebitis ( +) *N* = 313	Phlebitis (−) *N* = 3116	*P* value
Fentanyl (*n*, %)	1463 (13.5%)	10.6	49 (15.7%)	414 (13.3%)	0.24
Heparin (*n*, %)	334 (9.7%)	8.4	28 (8.9%)	306 (9.8%)	0.62
Fat (*n*, %)	308 (9.0%)	11.4	35 (11.2%)	273 (8.8%)	0.15
Nicardipine (*n*, %)	307 (9.0%)	16.9	52 (16.6%)	255 (8.2%)	< 0.0001
Dexmedetomidine (*n*, %)	292 (8.5%)	14.0	41 (13.1%)	251 (8.1%)	0.002
Ampicillin/sulbactam (*n*, %)	199 (5.8%)	10.1	20 (6.4%)	179 (5.7%)	0.64
Albumin (*n*,%)	176 (5.1%)	14.2	25 (8.0%)	151 (4.8%)	0.02
Paracetamol (*n*,%)	166 (4.8%)	6.0	10 (3.2%)	156 (5.0%)	0.16
Potassium (*n*, %)	154 (4.5%)	16.9	26 (8.3%)	128 (4.1%)	0.0006
Meropenem (*n*, %)	135 (3.9%)	11.9	16 (5.1%)	119 (3.8%)	0.26
Steroid (*n*, %)	125 (3.7%)	7.2	9 (2.9%)	116 (3.7%)	0.45
Ceftriaxone (*n*, %)	125 (3.7%)	10.4	13 (4.2%)	112 (3.6%)	0.62
Vancomycin (*n*, %)	120 (3.5%)	5.8	7 (2.2%)	113 (3.6%)	0.20
Magnesium (*n*, %)	111 (3.2%)	9.9	11 (3.4%)	100 (3.2%)	0.77
PPN (*n*, %)	92 (2.7%)	10.9	10 (3.2%)	82 (2.6%)	0.56
Phosphorus (*n*, %)	91 (2.7%)	13.2	12 (3.8%)	79 (2.5%)	0.17
Noradrenaline (*n*, %)	88 (2.6%)	21.6	19 (6.1%)	69 (2.2%)	< 0.0001
Carperitide (*n*, %)	88 (2.6%)	13.6	12 (3.8%)	76 (2.4%)	0.14
Midazolam (*n*, %)	60 (1.8%)	20.0	12 (3.8%)	48 (1.5%)	0.003
Nitroglycerin (*n*, %)	60 (1.8%)	10.0	6 (1.9%)	54 (1.7%)	0.81
Dobutamine (*n*, %)	50 (1.5%)	14.0	7 (2.2%)	43 (1.4%)	0.23
Cefmetazole (*n*, %)	48 (1.4%)	8.3	4 (1.3%)	44 (1.4%)	0.85
Amiodarone (*n*, %)	41 (1.2%)	22.0	9 (2.9%)	32 (1.0%)	0.004
Cefepime (*n*, %)	41 (1.2%)	9.8	4 (1.3%)	37 (1.2%)	0.89
Levetiracetam (*n*, %)	41 (1.2%)	26.8	11 (3.5%)	30 (1.0%)	< 0.0001
Landiolol (*n*, %)	40 (1.2%)	10.0	4 (1.3%)	36 (1.2%)	0.85

*PIVC* peripheral intravenous catheter, *PPN* peripheral parenteral nutrition

### Multivariable multilevel analysis for the occurrence of phlebitis

Univariable and multivariable multilevel marginal Cox regression analyses were performed to determine the risk factors for phlebitis (Table 4) after the Cox proportional hazards assumptions were checked for all risk factors (data were not shown). Spline curves of age and body mass index for the occurrence of phlebitis are shown in Additional file 1: Fig. S1. Age showed a linear association with phlebitis, but body mass index and APACHE II were not considered to be a linear effect on the occurrence of phlebitis. Therefore, the spline curves were used as a reference to set the cutoff values of body mass index (≤ 15, 16–22, 23–29, and 30 ≤) and APACHE II (≤ 15, 16–25, and 26 ≤). The drugs associated with increased incidence of phlebitis were nicardipine (HR, 1.85; 95% CI, 1.29–2.66), noradrenaline (HR, 2.42; 95% CI, 1.40–4.20), amiodarone (HR, 3.67; 95% CI, 1.75–7.71) and levetiracetam (HR, 5.65; 95% CI, 2.80–11.4). The multivariate analysis revealed that the only factor that increased the risk of developing phlebitis were drugs.

**Table 4:** Univariate and multivariable analysis for phlebitis using marginal Cox regression analysis for phlebitis

Variables	Univariable analysis *N* = 3429 Phlebitis: 313 (9.1%)		Multivariable analysis *N* = 2460 Phlebitis: 247 (10.0%)	
	HR (95% CI)	*p* value	HR (95% CI)	*p* value
ICU characteristics				
Drug administration standardization	0.36 (0.20–0.66)	0.0009	0.35 (0.17–0.76)	0.007
Education on venous catheter management for nurses	1.29 (1.03–1.63)	0.03	1.15 (0.86–1.54)	0.35
Patient characteristics				
Age	1.01 (0.99–1.01)	0.15	1.01 (0.99–1.02)	0.28
Gender, male	0.69 (0.55–0.86)	0.0009	0.85 (0.64–1.12)	0.24
BMI				
16–22	Ref.	-	Ref.	-
≤15	1.14 (0.45–2.90)	0.78	1.18 (0.60–2.33)	0.62
23–29	0.95 (0.62–1.46)	0.83	0.86 (0.65–1.14)	0.29
30≤	0.58 (0.14–2.38)	0.45	0.43 (0.20–0.95)	0.04
APACHE II				
16–25	Ref.	–	Ref.	–
≤15	1.09 (0.85–1.39)	0.49	1.07 (0.79–1.45)	0.65
25 <	0.77 (0.56–1.05)	0.10	0.70 (0.48–1.01)	0.06
Catheter characteristics				
Catheter inserted by (*n*,%)				
Nurse	Ref.	–	Ref.	–
Doctor	0.55 (0.35–0.86)	0.009	0.55 (0.32–0.94)	0.03
Number of trials for insertion (*n*,%)				
1	Ref.	–	Ref.	–
2	0.90 (0.60–1.34)	0.59	1.01 (0.67–1.53)	0.95
3	1.44 (0.89–2.32)	0.14	1.35 (0.81–2.24)	0.25
≥4	0.84 (0.35–2.04)	0.70	1.34 (0.54–3.33)	0.53
Use of ultrasonography	0.51 (0.19–1.38)	0.19	0.78 (0.24–2.56)	0.68
Inserted site				
Forearm	Ref.		Ref.	
Upper arm	0.61 (0.39–0.95)	0.03	0.52 (0.32–0.85)	0.009
Elbow	0.84 (0.50–1.43)	0.53	0.95 (0.50–1.79)	0.87
Wrist	0.70 (0.38–1.28)	0.24	0.56 (0.26–1.21)	0.14
Hand	0.58 (0.40–0.84)	0.004	0.67 (0.43–1.04)	0.08
Lower leg	0.87 (0.56–1.34)	0.52	0.74 (0.46–1.21)	0.23
Dorsal foot	0.96 (0.58–1.57)	0.86	0.95 (0.55–1.66)	0.87
Catheter size				
22–24G	Ref.		Ref.	
≥18G	0.99 (0.60–1.61)	0.96	1.97 (0.68–5.77)	0.21
20G	0.59 (0.44–0.79)	0.0004	0.75 (0.52–1.08)	0.12
Dressing				
Sterile dressing	Ref.		Ref.	
Non-sterile dressing	1.36 (0.70–2.64)	0.36	0.90 (0.28–2.89)	0.86
Catheter material				
Polyurethane	Ref.		Ref.	
PEU-Vialon*	0.61 (0.46–0.81)	0.0007	0.70 (0.49–1.02)	0.06
Tetrafluoroethylene	0.84 (0.64–1.09)	0.18	0.99 (0.71–1.38)	0.95
Others	1.37 (0.72–2.62)	0.34	0.94 (0.21–4.16)	0.93
Infection during catheter dwell	1.10 (0.86–1.40)	0.47	1.36 (0.99–1.85)	0.06
Drug characteristics				
Fentanyl (*n*, %)	0.89 (0.65–1.21)	0.45	0.81 (0.54–1.21)	0.29
Heparin	0.62 (0.42–0.92)	0.02	0.65 (0.40–1.07)	0.09
Fat	1.89 (1.04–3.41)	0.04	0.67 (0.40–1.10)	0.11
Nicardipine	1.97 (1.46–2.65)	< 0.0001	1.85 (1.29–2.66)	0.0008
Dexmedetomidine	1.16 (0.83–1.61)	0.38	1.08 (0.72–1.63)	0.70
Ampicillin/sulbactam	1.00 (0.63–1.57)	0.99	0.79 (0.47–1.31)	0.36
Albumin	0.87 (0.44–1.71)	0.68	1.36 (0.82–2.23)	0.23
Paracetamol	0.63 (0.34–1.18)	0.15	0.69 (0.33–1.46)	0.33
Potassium	1.62 (1.08–2.42)	0.02	1.32 (0.79–2.21)	0.30
Meropenem	0.76 (0.46–1.26)	0.28	0.99 (0.52–1.87)	0.96
Steroid	0.62 (0.32–1.20)	0.16	0.68 (0.32–1.43)	0.31
Ceftriaxone	0.88 (0.51–1.54)	0.66	0.69 (0.37–1.31)	0.26
Vancomycin	0.42 (0.20–0.87)	0.02	0.43 (0.18–1.03)	0.06
Magnesium	0.98 (0.54–1.79)	0.95	0.68 (0.27–1.69)	0.40
PPN	0.90 (0.48–1.69)	0.74	0.78 (0.37–1.62)	0.50
Phosphorus	1.30 (0.73–2.31)	0.38	1.11 (0.53–2.33)	0.79
Noradrenaline	2.54 (1.60–4.04)	< 0.0001	2.42 (1.40–4.20)	0.002
Carperitide	1.13 (0.64–2.02)	0.67	0.93 (0.46–1.84)	0.83
Midazolam	1.63 (0.92–2.91)	0.10	1.50 (0.72–3.11)	0.28
Nitroglycerin	0.73 (0.32–1.63)	0.44	0.22 (0.05–0.92)	0.04
Dobutamine	1.54 (0.73–3.26)	0.26	1.14 (0.48–2.68)	0.77
Cefmetazole	1.20 (0.45–3.22)	0.72	0.92 (0.22–3.82)	0.90
Amiodarone	2.34 (1.21–4.55)	0.01	3.67 (1.75–7.71)	0.0006
Cefepime	0.58 (0.22–1.56)	0.28	0.70 (0.25–1.96)	0.49
Levetiracetam	1.54 (0.21–11.1)	0.67	5.65 (2.80–11.4)	< 0.0001
Landiolol	0.75 (0.28–2.00)	0.56	0.73 (0.23–2.38)	0.61

Akaike’s information criterion, 3476.5

*APACHE* Acute Physiology And Chronic Health Evaluation, *BMI* body mass index, *CI* confidence interval, *ICU* intensive care unit, *HR* hazard ratio, *PPN* peripheral parenteral nutrition

On the other hand, factors that had a significantly decreased association with phlebitis were standardized drug administration measures in the ICU (HR, 0.35; 95% CI, 0.17–0.76), 30≤ BMI (HR, 0.43; 95% CI, 0.20–0.95), a catheter inserted by a doctor as nurse reference (HR, 0.55; 95% CI, 0.32–0.94), and upper arm insertion site as forearm reference (HR, 0.52; 95% CI, 0.32–0.85). Nitroglycerin was associated with decreased phlebitis (HR, 0.22; 95% CI, 0.05–0.92). Furthermore, both univariable and multivariable regression models showed consistent estimates for HRs and suggested the robustness of the analyses.

The sensitivity analysis results using the backward selection method in the multivariate analysis are shown in Additional file 1: Table S5. The results were similar to those of the primary analysis.

## Discussion

This study examined and identified the risk factors associated with phlebitis in critically ill patients admitted to the ICU. In particular, in the final multivariate model, the standardized drug administration measures in the ICU, use of the upper arm for catheter insertion, and nitroglycerin were shown to have a significantly reduced association with phlebitis. However, the most common factors that increased the incidence of phlebitis were drugs, such as nicardipine, noradrenaline, amiodarone, and levetiracetam. Some findings of this study pertaining to critically ill patients were similar to the findings of studies in a general hospital ward setting [10–12, 19–30]. Previous studies related to general wards showed the increased risk of phlebitis with the administration of nicardipine and noradrenaline [12, 31–33]. In addition, the present study showed that PIVC insertion in the upper arm was significantly associated with lower phlebitis rates than that in the forearm, similar to a finding reported in previous studies in general wards [34].

This study found that standardized drug administration measures in the ICU may have reduced the risk of phlebitis. Although there have been many reports on the various benefits of pharmacist interventions in the ICU [35–37], no study has examined the impact of specific interventions, such as standardized drug administration measures in the ICU. The role of pharmacists in improving the quality of care in the ICU has been clearly defined by the Society of Critical Care Medicine (SCCM) [38]. The SCCM recommends that ICU pharmacists monitor the appropriateness of drug administration, including the regimen used and potential for drug interactions, but the method was not indicated. The standardized drug administration measures in the ICU in this study was defined in detail according to documented standard operating procedures for drug administration. Thus, even if a drug with a high potential for causing complications is administered according to the predetermined rules, the risk of phlebitis can be reduced as much as possible. Although potassium is generally thought to increase the risk of phlebitis [39], it was not identified in this study as a factor that increased the risk of phlebitis, likely as our protocols for potassium administration in the ICU involve only low doses through a PIVCs. Along with using the expertise of ICU pharmacists, establishing predetermined rules on how the drugs are administered may help reduce the incidence of phlebitis.

Few reports have assessed the risk of phlebitis from PIVCs inserted into the upper extremities, and the risk remains unknown. Similar to midline catheters and peripherally inserted central catheters (PICCs) inserted into the upper extremities [40], PIVCs inserted in the upper arm are generally placed in large-diameter vessels to facilitate natural hemodilution of IV drugs and reduce the likelihood of chemical phlebitis [41]. This could be attributed to the following mechanisms: first, the mechanisms underlying phlebitis is mechanical stimulation of the vascular wall by catheters [42, 43], and catheters inserted in highly mobile areas, such as the hand and forearm may cause phlebitis due to the increased stress on the vascular wall if the catheter is not firmly fixed [44]. Second, the large diameter of the veins in the upper arm may lead to a reduced risk of phlebitis. If the diameter of the peripheral venous catheter is large relative to the diameter of the blood vessel, the stimulus hitting the vessel wall may be increased. Studies have shown that the rate of catheter complications is higher in the upper-extremity veins than in the lower-extremity veins [34], suggesting that the size of the vessel diameter contributes to the development of phlebitis.

A large number of drugs are administered to critically ill patients in the ICU; therefore, the frequency of PIVC-related complications is expected to be high. Various studies have reported the effect of administered drugs on the development of phlebitis [32, 33, 39, 45, 46]. In the present study, the effects of 26 drugs on phlebitis selected from a database of more than 300 drugs were examined. It is recommended that high-risk drugs for PIVC-related complications, such as nicardipine and noradrenaline, be administered through a central venous catheter. However, because of complications associated with central venous catheters [47, 48], such drugs are often administered through PIVCs. There is still uncertainty about the best device selection when administering those drugs with high phlebitis risk. This study may provide new insights into PIVC management, and these considerations could be used for device selection.

This study had limitations. First, we could not examine the risk factors for phlebitis by its severity and only examine risk factors for low-grade phlebitis. The severity of phlebitis included in this analysis was mostly grade 1 (73.8%), and grade 3 and 4 together accounted for only 4.5%. Therefore, whether the study results could be applied to more severe phlebitis is unknown. Second, it was impossible to verify whether the multivariate model presented in this study was the best one. In this study, only clinically important factors for phlebitis development were included in the multivariate analysis, and model selection methods such as the stepwise method were not used. This may have resulted in underestimation or overestimation of each potential risk factor. However, since this was exploratory research of risk factors and not a predictive modeling study, the multivariate model was believed to have achieved the minimum objective. In addition, the present study was conducted as multivariate analysis (exploratory analysis), making it impossible to show a causal relationship between each factor and phlebitis occurrence. Third, not all the drugs administered could be included in the multivariable analysis. However, there may have been other drugs besides the selected ones that posed a risk of phlebitis development. It was difficult to examine all the drugs using a classical multivariable model, and further investigation may be necessary to explore the risk of phlebitis caused by all the drugs. Fourth, the drugs included in the multivariable analysis of this study were binary variables, which may not have correctly assessed the inherent risk of the drugs. The drug effect depends not only on its administration but also on the drug dose administered. Hence, there is a need to assess the risk of drugs causing phlebitis using methods that can assess the risk of the administered drug, other than the binary variable method. Therefore, the study results may not have direct clinical relevance. It may be necessary to evaluate the risk of drugs as a continuous variable. Finally, the present analysis method does not address the issue of time-dependent confounding of drug administration, leaving immortal time bias. The time zero for COX regression analysis is ICU admission, and the time lag before the drug is administered may cause immortal time bias. A major reason that this study considered drug administration variables as a binary variable of whether the drug was administered was the difficulty in extracting the timing of initiation of each drug from patient charts at all 22 participating facilities. Another reason is that this study assumed it would be best to treat the drugs as a binary variable as the first used in this study to compare the results of this study with those of previous studies. This study also believes it is necessary to conduct analyses that consider time-dependent variables; therefore, this will be the subject of the next study.

## Conclusions

Various factors may be involved in the phlebitis development caused by peripheral venous catheters in critically ill patients, including institutional, patient, catheter, and drug-induced factors. The involvement of drug factors is particularly important for phlebitis caused by peripheral venous catheters in critically ill patients. Further investigation that can examine many drug factors simultaneously and the evaluation of drug factors is necessary.

## Supplementary Information

**Additional file 1:** **Table S1.** The definition of phlebitis (INS). **Table S2.** The definition of each element of INS phlebitis definition. **Table S3. **Proportion and incidence of phlebitis per catheter. **Table S4.** Characteristics of phlebitis. Table S5. Sensitivity analysis of multivariable analysis for phlebitis using backward selection method. **Figure S1.** Spline curve of age and body mass index for the Occurrence of Phlebitis. **a** age, **b** body mass index, **c** APACHE II.

**Additional file 2.** STROBE Statement—checklist of items that should be included in reports of observational studies

## Data Availability

The datasets generated during and/or analyzed during the current study are not publicly available due to post hoc analyses by the co-authors. However, they are available from the corresponding author on reasonable request.

## Abbreviations

APACHE: Acute Physiology and Chronic Health Evaluation
CI: Confidence interval
ER: Emergency room
HR: Hazard ratio
ICU: Intensive care unit
INS: Infusion Nurse Society
IQR: Interquartile range
PICC: Peripherally inserted central catheter
PIVC: Peripheral intravascular catheters
SAPS: Simplified acute physiology score
SCCM: Society of Critical Care Medicine
SD: Standard deviation
SOFA: Sequential organ failure assessment
