# Supplementary material for: Risk factors for peripheral intravascular catheter-related phlebitis in critically ill patients: analysis of 3429 catheters from 23 Japanese intensive care units
Source: Ann Intensive Care. 2022 Apr 8;12:33. doi: 10.1186/s13613-022-01009-5 (PMC8994002; doi:10.1186/s13613-022-01009-5)
Supplement: Supplementary file 1 — Additional file 1: Table S1. The definition of phlebitis (INS). Table S2. The definition of each element of INS phlebitis definition. Table S3. Proportion and incidence of phlebitis per catheter. Table S4. Characteristics of phlebitis. Table S5. Sensitivity analysis of multivariable analysis for phlebitis using backward selection method. Figure S1. Spline curve of age and body mass index for the Occurrence of Phlebitis. a age, b body mass index, c APACHE II. [file 13613_2022_1009_MOESM1_ESM.docx]

**Additional file 1**

**Data collection item in original study**

*ICU Characteristics*

We distributed a key informant survey at each site to collect data on the ICU characteristics, such as the number of beds in the ICU, provision of education on venous catheter management, presence of regular catheter replacement, presence of an established definition of phlebitis and catheter-related blood stream infection in the ICU, type of ICU patients (surgical, medical, or mixed), number of full-time equivalent ICU physicians per day, standardized drug administration measures in the ICU (standardized education on drug administration and combination contraindication tables), presence of a pharmacist in the ICU (e.g., full-time or daytime), and presence of nurses with specialized education on managing venous catheters at each institution (named IV nurse; no specific definition of specialized education or IV nurse was employed).

*Patient Characteristics*

Data collected included patient demographics, medical histories (Charlson comorbidity index [1], hypertension, and dyslipidemia), ICU admission routes (emergency department, ward, or transfer from other hospital), disease types at ICU admission (elective surgical, emergency surgical, and medical), presence of sepsis at ICU admission (defined by Sepsis-3 criteria [2]), length of ICU stays, and length of hospital stays. The following data were collected within the first 24 hours after ICU admission: the vital signs and laboratory data needed for the calculation of Acute Physiology and Chronic Health Evaluation (APACHE) II scores [3], Simplified Acute Physiology Scores (SAPS) II [4], and the Sequential Organ Failure Assessment (SOFA) scores [5].

*Catheter Characteristics*

The following variables were collected for all the PIVCs, regardless of the insertion location: insertion dates, removal dates, insertion sites, places of insertions and removals (in the ICU or outside the ICU), catheter materials (description of trade name), catheter gauges, dressing methods (gauze, non-sterile dressing, sterile dressing, or chlorhexidine-impregnated dressing), presence of any types of infections during the catheter dwell. Furthermore, when the PIVCs were inserted in the ICU, additional data were collected. These data included the medical staff inserting the catheter, the number of punctures, skin antiseptics, types of gloves (non-sterile or sterile), and difficulties with the insertions. In addition, with regard to the insertion site of the PIVC, the puncture site was described every four hours (the details are described in the Outcome section). Catheter information was recorded only during the patient’s ICU stay.

*Drug Characteristics*

We gathered all the information related to the drugs administered through each PIVC, excluding catheter lock solutions such as heparin and saline. The information included the drug composition, drug concentrations, starting times of drug administration, times that the rate of the drug being administered was changed, rate of drug administration, and the times that the drug being administered was stopped. The drug data were obtained from the electronic medical records and sent to the data management center. Drug information was recorded only during the ICU stay.

**Definition and measurement of phlebitis**

The diagnosis of phlebitis was made by a blinded assessor at the main facility, according to criteria that comprised six clinical signs, and was classified into four grades (e-Table 1, see Additional File 1). If patients could not assess their symptoms such as pain, well-trained nurses (defined in each study institution) assessed the grade of pain using such as face scale and behavior pain scale. To reduce information bias, pilot training was conducted to enable accurate phlebitis diagnosis. Furthermore, the accuracy of phlebitis diagnosis was monitored during the study period at the central institution by a well-trained, expert clinician researcher. The accuracy of the information on the catheter insertion sites was confirmed by sending phlebitis images to the data management center from each study institution for the first month after commencing data collection.

References

1. Charlson ME, Pompei P, Ales KL, MacKenzie CR. A new method of classifying prognostic comorbidity in longitudinal studies: development and validation. J Chronic Dis. 1987;40:373-83.

2. Seymour CW, Liu VX, Iwashyna TJ, Brunkhorst FM, Rea TD, Scherag A, et al. Assessment of Clinical Criteria for Sepsis: For the Third International Consensus Definitions for Sepsis and Septic Shock (Sepsis-3). JAMA. 2016;315:762-74.

3. Knaus WA, Draper EA, Wagner DP, Zimmerman JE. APACHE II: a severity of disease classification system. Crit Care Med. 1985;13:818-29.

4. Le Gall JR, Lemeshow S, Saulnier F. A new Simplified Acute Physiology Score (SAPS II) based on a European/North American multicenter study. JAMA. 1993;270:2957-63.

5. Vincent JL, Moreno R, Takala J, Willatts S, De Mendonca A, Bruining H, et al. The SOFA (Sepsis-related Organ Failure Assessment) score to describe organ dysfunction/failure. On behalf of the Working Group on Sepsis-Related Problems of the European Society of Intensive Care Medicine. Intensive Care Med. 1996;22:707-10.

**Table S1 The definition of phlebitis (INS)**

| Grade | Criteria |
| --- | --- |
| 0 | No clinical symptoms |
| 1 | Erythema at access site with or without pain |
| 2 | Pain at access site with erythema and/or edema |
| 3 | Pain at access site with erythema and/or edema, streak formation, palpable venous cord |
| 4 | Pain at access site with erythema and/or edema, or palpable venous cord > 1 inch, purulent drainage |

INS, infusion nurse society

**Table S2 The definition of each element of INS phlebitis definition**

| Element | definition |
| --- | --- |
| Pain | Pain around PIVC insertion site |
| Erythema | Redness around PIVC insertion site |
| Edema | Swelling around PIVC insertion site |
| Streak formation | Redness along blood vessels at PIVC insertion site |
| Palpable venous cord | Induration along blood vessels at PIVC insertion site |

INS, infusion nurse society; PIVC, peripheral intravenous catheter

**Table S3 Proportion and incidence of phlebitis per catheter**

| Phlebitis per catheter | |
| --- | --- |
| Number of catheters/total PIVCs | 313/3,429 |
| Proportion, % (95% CI) | 9.1 (8.2-10.1) |
| Time to phlebitis*, median (IQR), hours | 37.0 (19.2-57.6) |
| Incidence rate per 100 intravenous catheter days (95% CI) | 3.5 (3.1-3.9) |

CI, confidence interval; CRBSI, catheter-related blood stream infection; ICU, intensive care unit; IQR, interquartile range; PIVC, peripheral intravenous catheter

*time to phlebitis means the duration between insertion of the catheter and detection of phlebitis.

**time to removal means the duration between insertion and removal of the catheter.

**Table S4 Characteristics of phlebitis**

| Variables | | |
| --- | --- | --- |
| Variables | | Total  n=313 |
| No. of times phlebitis observed before removal (n, %) | |  |
| 1 | | 224/313 (71.6%) |
| 2 | | 36/313 (11.5%) |
| 3 | | 16/313 (5.1%) |
| 4 | | 10/313 (3.2%) |
| 5 | | 18/313 (5.6%) |
| >5 | | 9/313(2.9%) |
| Maximal grade of phlebitis | |  |
| 1 | (n, %) | 231/313 (73.8%) |
|  | Time to removal*, median (IQR), hours | 37.1 (19.1-59.5) |
| 2 | (n, %) | 68/313 (21.7%) |
|  | Time to removal*, median (IQR), hours | 31.1 (18.0-55.3) |
| 3 | (n, %) | 9/313 (2.9%) |
|  | Time to removal*, median (IQR), hours | 23.4 (18.7-62.0) |
| 4 | (n, %) | 5/313 (1.6%) |
|  | Time to removal*, median (IQR), hours | 38.5 (36.4-47.9) |

CI, confidence interval; CRBSI, catheter-related blood stream infection; ICU, intensive care unit; IQR, interquartile range; PIVC, peripheral intravenous catheter

*time to removal means the duration between insertion and removal of the catheter.

Table S5 Sensitivity analysis of multivariable analysis for phlebitis using backward selection method

| variables | Multivariable analysis  N=2,422  Phlebitis: 241 (10.0%) | |
| --- | --- | --- |
|  | HR (95% CI) | p value |
| ***ICU characteristics*** |  | |
| Drug administration standardization | 0.45 (0.22-0.93) | 0.03 |
| ***Patient characteristics*** |  | |
| BMI |  |  |
| 16-22 | ref | - |
| ≦15 | 1.57 (0.82-2.99) | 0.17 |
| 23-29 | 0.89 (0.68-1.17) | 0.42 |
| 30≦ | 0.40 (0.19-0.86) | 0.02 |
| ***Catheter characteristics*** |  |  |
| Catheter inserted by (n,%) |  |  |
| Nurse | ref | - |
| Doctor | 0.58 (0.35-0.94) | 0.03 |
| ***Drug characteristics*** |  | |
| Fat | 0.64 (0.41-0.99) | 0.046 |
| Nicardipine | 1.95 (1.40-2.72) | <0.0001 |
| Dexmedetomidine | 1.08 (0.72-1.63) | 0.70 |
| Piperacillin/sulbactam | 0.23 (0.09-0.55) | 0.001 |
| Noradrenaline | 2.83 (1.70-4.71) | <0.0001 |
| Nitroglycerin | 0.21 (0.05-0.87) | 0.03 |
| Amiodarone | 2.90 (1.42-5.90) | 0.003 |
| Levetiracetam | 3.89 (2.04-7.41) | <0.0001 |

Akaike’s Information Criterion; 3341.7

The factors included in the backward selection method are as follows; age, albumin, any infection during catheter dwell, ampicillin/sulbactam, antiseptic solution before catheterization, acute physiology and chronic health evaluation, carperitide, catheter gauge, catheter material, cefepime, cefmetazole, ceftriaxone, dexmedetomidine, difficulties with the insertions, dobutamine, dressing, fentanyl, gender, grove, heparin, ICU admission route, inserted site, landiolol, magnesium, meropenem, midazolam,number of trials for insertion, paracetamol, peripheral parenteral nutrition, phosphorus, potassium, sepsis at ICU admission, steroid, use of ultrasonography, vancomycin

BMI, body mass index; CI, confidence interval; ICU, intensive care unit; HR, hazard ratio

Figure S1 Spline Curve of age and body mass index for the Occurrence of Phlebitis. (a) age, (b) body mass index, (c) APACHE II
